# A new reporter design based on DNA origami nanostructures for quantification of short oligonucleotides using microbeads

**DOI:** 10.1038/s41598-019-41136-x

**Published:** 2019-03-18

**Authors:** Youngeun Choi, Carsten Schmidt, Philip Tinnefeld, Ilko Bald, Stefan Rödiger

**Affiliations:** 1 University of Potsdam, Department of Chemistry, Physical Chemistry, 14476 Potsdam, Germany; 2 BAM Federal Institute for Materials Research and Testing, 12489 Berlin, Germany; 3 Brandenbrug University of Technology Cottbus-Senftenberg, Institute of Biotechnology, 01968 Senftenberg, Germany; 4 Department Chemie and Center for NanoScience, Ludwig-Maximilians-Universitaet Muenchen, Butenandtstr, 5-13 Haus E, 81377 Muenchen, Germany

## Abstract

The DNA origami technique has great potential for the development of brighter and more sensitive reporters for fluorescence based detection schemes such as a microbead-based assay in diagnostic applications. The nanostructures can be programmed to include multiple dye molecules to enhance the measured signal as well as multiple probe strands to increase the binding strength of the target oligonucleotide to these nanostructures. Here we present a proof-of-concept study to quantify short oligonucleotides by developing a novel DNA origami based reporter system, combined with planar microbead assays. Analysis of the assays using the VideoScan digital imaging platform showed DNA origami to be a more suitable reporter candidate for quantification of the target oligonucleotides at lower concentrations than a conventional reporter that consists of one dye molecule attached to a single stranded DNA. Efforts have been made to conduct multiplexed analysis of different targets as well as to enhance fluorescence signals obtained from the reporters. We therefore believe that the quantification of short oligonucleotides that exist in low copy numbers is achieved in a better way with the DNA origami nanostructures as reporters.

## Introduction

Development of nucleic acid detection systems has increased rapidly over the past few years, with the discovery of potential biomarkers for early diagnosis of diseases such as cancer or type II diabetes^1^. A variety of detection systems already exists, ranging from electrochemical methods to optical methods. Many of these systems rely on the specific hybridisation between targets and the probe^2^. Although such progress has been made in nucleic acid detection, due to newly emerging biomarkers such as circulating DNA, mRNA, and microRNA (miRNA) continuous efforts are required to develop detection methods that not only have improved limits of detection but also allow for highly multiplexed analysis^3^.

For example, miRNAs are short noncoding RNA molecules approximately 18–24 nucleotides in length and play an important role in gene regulation^4^. Of interest is their potential as biomarkers for cancer^5,6^, ever since it was shown that their expression levels are aberrant in chronic lymphocytic leukaemia^7^. This has encouraged many studies to develop profiling methods especially with regard to multiplexed analysis and detection without amplification^8^.

For the detection and profiling of such short target nucleic acids, it is essential to have a competent reporting method. Not only is the absolute brightness important but the binding kinetics to the target is also a critical aspect of developing a good reporter. A programmable, self-assembled DNA origami nanostructure^9^ that is spatially addressable can be utilised to design an elegant reporter system with the aforementioned qualities. They can be thought of as breadboards in the nanoscale, as the oligonucleotides used in this nanostructure are individually addressable. This leads to a highly versatile nanostructure where many functionalities such as organic dyes^10–12^, quantum dots^13^, nanoparticles, and proteins^14,15^ can be placed. The simple synthesis, high versatility, and the ability to place molecules with spatial control makes these DNA nanostructures a good platform to develop reporters with multiple chromophores for signal enhancement as well as binding sites for targets.

Another aspect to consider when profiling bioanalytes at low concentration is the detection method. Assays based on microbeads are a powerful technique that can achieve a low limit of detection (LoD) as well as it is capable of multiplexing. Microbead-based assays provide many advantages compared to conventional analytical techniques such as smaller sample consumption, high degree of multiplexing, and high throughput capability^16^. Accordingly, they have been used extensively in bioanalysis including single nucleotide polymorphisms^17^, standard PCR products^18^, miRNA^19^, pathogens^20^, as well as reactions such as *in vitro* cloning of DNA^21^, aptamer generation^22^, and gene expression analysis^23^. Such vast bioanalytical applications of microbeads stems from the spherical structure of the beads providing higher surface area compared to planar structures of a microarray. This allows the reactions on microbead surfaces to be similar to reactions in solution bringing the sensitivity of the array down to the zeptomolar range^24^. Multiplex analysis can be realised by encoding the beads through incorporation of two or more organic dyes in various ratios, thus assigning each bead with a unique fluorescence signature^18^. Each particle therefore can be thought of as a spot on a microarray, where reactions and detections can be conducted independently, with each population of the microbead assigned to one analyte only^25^.

Of the many strategies for the detection of microbeads including flow cytometry^26,27^, microfluidics^28,29^, and image based techniques^30,31^, a microscopy based technique termed ‘VideoScan’ provides a method of identifying and measuring randomly ordered microbeads^32^. It is a very versatile platform which uses standard equipment and consumables, and can be integrated with heating and cooling units, allowing it to be easily adaptable to the user’s needs. This technique has been successfully applied in bioanalysis such as detection of nucleic acids^32^, autoimmune diagnostics^33^, epitope mapping^34^, and serotyping of *E. coli*^35^ as well as quantifying carboxyl groups on a microbead surface^36^.

In this work, we present a reporter design based on DNA origami nanostructures to detect short oligonucleotides in a microbead-based assay. DNA analogues of poly adenosine extended miRNA have been chosen as target sequences for this proof-of-concept study, as the stability of DNA-DNA duplexes are comparable with that of DNA-RNA duplexes^37^. Multiple organic dyes as well as hybridisation-based target binding sites are strategically placed on the DNA origami nanostructure and their response to the target is detected using VideoScan. A comparison between this new type of reporter and a simple single stranded DNA labelled with one dye molecule is done by determining assay parameters for each system, thereby showing the potential of the newly designed reporter for improved miRNA detection.

## Results

### DNA origami nanostructure design

**Figure 1:**
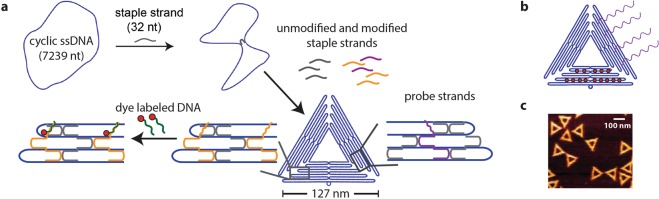
DNA origami reporter design. (**a**) DNA origami folding steps with the first step to fold the triangular structure with probe strands to capture the target and handles for dye labeled DNA. In the subsequent hybridisation step, the dye labeled DNAs are positioned on the predetermined locations on the DNA origami. (**b**) DNA origami reporter design with 14 dye molecules (red circles) placed with approximately 11 nm gap on one trapezoid and four probe strands (purple lines) on a different trapezoid of the triangular DNA origami nanostructure. (**c**) AFM image of the folded triangular DNA origami nanostructures (scale bar 100 nm) with a height profile of approximately 1.8 nm.

DNA origami nanostructures provide the platform to design a bright and versatile reporter system. As shown in Fig. 1a, DNA origami nanostructures are folded containing staple strands used as handles to place dye molecules as well as probe strands to hybridise to the target oligonucleotide. In this DNA origami, 14 dye capture strands are positioned on one of the trapezoids of the triangle with which after subsequent hybridisation with an ATTO 647N labelled complementary DNA, they have a distance of 11–12 nm apart from each other (with an inter-helix gap of 1 nm). This spatial arrangement prevents self-quenching of the ATTO 647N dyes^38^ that can happen due to dye-aggregation^39^. Probe strands are positioned on a different trapezoid of the triangular DNA origami nanostructure than where the dye labels are positioned, as well as on the opposite plane of the triangle as to the dye labelling strands. For the DNA origami design with four probe sites, the gap between each probe strand is approximately 22 nm which provides sufficient space for a single stranded target DNA to hybridise without the hybridisation yield being affected^40,41^. Schematic representation of such a DNA origami reporter with 14 dye molecules and four probe strands is shown in Fig. 1b, and their triangular structure can be confirmed after folding by AFM imaging (Fig. 1c). Microbead-based assay using VideoScan technology.

**Figure 2:**
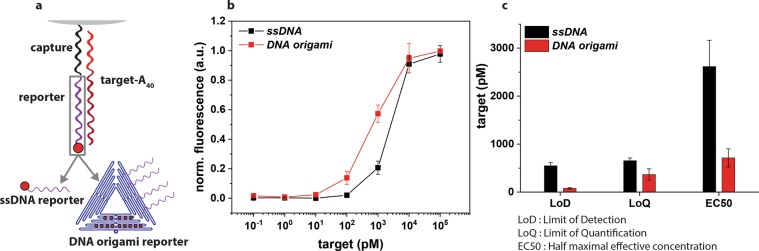
Comparison of the two reporters for target detection; single stranded oligonucleotide (*ssDNA*) and DNA origami (*DNA origami*). (**a**) Microbeads are functionalised with streptavidin to which biotinylated capture DNAs (20 bases, target oligonucleotide specific) are immobilised. Target oligonucleotides extended with an A_40_ handle are subsequently hybridised, thus immobilising the analyte of interest to the surface of the microbead. Finally, the reporters (labeled with ATTO 647N) which have a probe sequence of T_40_ are hybridised with the target. (**b**) Normalised surface fluorescence signal on microbeads for *ssDNA* (black squares) and *DNA origami* (red squares) according to the target oligonucleotide concentration with each reporter at 2.5 nM. Two different microbead populations were used for both assays. (**c**) Assay parameters such as LoD, LoQ, and EC50 quantified from four individual dilution series for each reporter. By using *DNA origami*, the LoD and LoQ can be shifted towards a lower concentration of the target oligonucleotide.

Size- and colour-coded microbeads are prepared for detection of target oligonucleotides by first immobilising a target specific capture DNA on the surface, then subsequently hybridising the target oligonucleotide. This target contained a 20 nt sequence with a sticky end (Poly(A)_40_) that acts as the handle for the dye-labelled reporters to immobilise on the surface of the microbeads. Extending the short oligonucleotide with a poly adenosine end is a labelling method which is quite common for detecting miRNA^42^, and is also beneficial for our detection method. The surface fluorescence intensity generated by these reporters was then measured using VideoScan (Fig. 2). The response of two different reporters, a conventional single stranded DNA labelled with one dye molecule (*ssDNA*), and a DNA origami nanostructure reporter (*DNA origami*, Fig. 1b) were compared as shown in Fig. 2b, both showing an increase in the fluorescence intensity until reaching maximum intensity at 10 nM target concentration. Several key assay performance characteristics were quantified (Fig. 2c) for the two reporters such as LoD, limit of quantification (LoQ), and half maximal effective concentration (EC50)^43^. *DNA origami* showed a lower LoD (78 pM), as well as a lower LoQ (366 pM) for the assay making it a more sensitive reporter for the detection of targets at lower concentrations than when using *ssDNA* as a reporter. In addition, the dynamic range was larger for *DNA origami* (0.1–10 nM) than for *ssDNA* (1–10 nM). These parameters show that by using *DNA origami*, the assay can be used to quantify target oligonucleotides not only at a lower concentration but also at a wider concentration range than when using a simple *ssDNA*.

### Proof-of-concept for multiplexed analysis

**Figure 3:**
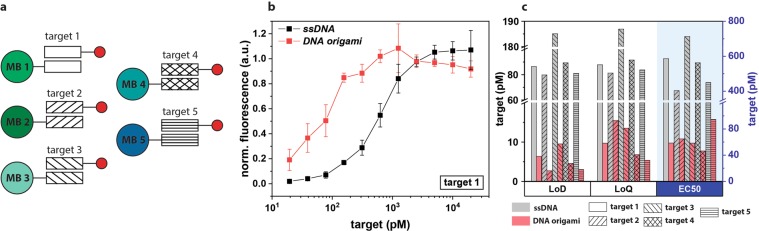
Multiplex analysis. (**a**) Schematic representation of five different target oligonucleotides tested for multiplex analysis. Each target sequence is assigned to one microbead population with different fluorescent codes (MB n, n = 1–5). Red circle represents the reporter. (**b**) Normalised surface fluorescence signal on microbead for *ssDNA* (black squares) and *DNA origami* (red squares) according to target 1 concentration with each reporter at 2.5 nM. (**c**) Assay parameters (LoD, LoQ, EC50) determined from signal response plots for all targets all show similar values. As shown in Fig. 2c, clear distinction between *ssDNA* and *DNA origami* was shown with *DNA origami* showing significantly lower values for all three parameters (EC50 values shown on right axis).

By using microbeads that are encoded with different ratios of fluorescent dyes, each analyte of interest can be assigned to a specific microbead population thus allowing for multiplex analysis. This is a simple but very powerful technique which has been used widely in multiplexed microbead-based assays^18,44^. Here, five different target oligonucleotide sequences all extended with an A_40_ sticky end and five different microbead populations were chosen for the proof-of-concept for multiplex analysis (Fig. 3). Once all the microbeads were functionalised with target capture, target, and reporter, the five microbead populations were mixed together in one microwell for VideoScan measurements. Both reporters showed similar trends for all the targets, with *DNA origami* reaching maximum intensity at lower concentration than for *ssDNA* as is shown in Fig. 3b for target sequence 1. For all five targets, *DNA origami* showed a dynamic range more suitable for detection of lower target concentration than *ssDNA*. The determined assay parameters LoD, LoQ, and EC50 are shown in Fig. 3c, with *DNA origami* showing lower values for all parameters (LoD; 2.7–9.5 pM, LoQ; 5.3–15.5 pM, EC50; 46–94 pM) than *ssDNA* (LoD; 80–185 pM, LoQ; 81–186 pM, EC50; 403–713 pM). This is mainly attributed to a higher hybridisation efficiency of *DNA origami* due to multiple possible binding sites in conjunction with the higher number of dyes per reporter.

### Improving the measured signal

By using DNA origami, it has been shown in previous sections that the microbead-based assay can be optimised for detection of lower target oligonucleotide concentration which is beneficial for the ultimate detection target of miRNA with low copy numbers. Although multiple dye molecules are present on the *DNA origami* probes, the absolute fluorescence intensity is lower than expected (Fig. S-1). This could be explained by the simple fact that the *DNA origami* is larger than *ssDNA* and therefore blocking free targets that are available for binding to reporters. Another explanation for the low signal intensity could be due to the hybridisation step of the reporter being far more complex than expected, making it difficult for the reporters to bind efficiently to the microbead.

**Figure 4:**
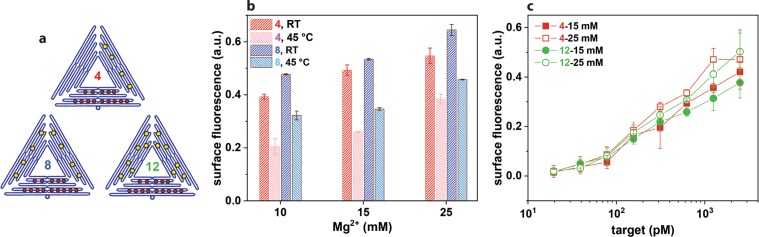
Effect of the number of probe strands and binding conditions on signal intensity. (**a**) Schematic representation of DNA origami with 4 (red), 8 (blue), 12 (green) probe strands. (**b**) Surface fluorescence measured for DNA origami with 4 probe strands at room temperature (red) and at 45 °C (pink), and with 8 probe strands at room temperature (blue) and at 45 °C (light blue) all at three different Mg^2+^ concentrations (at 100 nM target oligonucleotide). (**c**) Surface fluorescence measured according to the target concentration for DNA origami with 4 probe strands at 15 mM Mg^2+^ (), at 25 mM Mg^2+^(), and 12 at 15 mM Mg^2+^ (), and at 25 mM Mg^2+^().

To improve the signal-to-noise ratio, the effect of the binding strength of the DNA origami to the microbead (with the target oligonucleotides already immobilised on the microbeads) on the signal intensity was observed. The hybridisation conditions, i.e. temperature and amount of magnesium ion in the hybridisation solution, were varied (Fig. 4b) for *DNA origami* with 4 and 8 probe strands. A clear decrease in the fluorescence intensity was observed by raising the hybridisation temperature to 45 °C compared to hybridisation at room temperature (RT) indicating a correlation between the hybridisation efficiency and the recorded fluorescence intensity. In addition, with an increase in the magnesium concentration in the hybridisation solution from 10 mM to 25 mM, a slight increase in the fluorescence intensity was shown. This is due to the increase in ionic strength providing more screening of the electrostatic repulsion between the negatively charged backbone of the DNA, and thus increasing the melting temperature^45,46^. It was hypothesised that an increase in the number of probe strands provides stronger binding between the reporter and the target, and in Fig. 4b, a slight increase in the fluorescence signal was observed for *DNA origami* with 8 probes strands. Based on these results, a concentration dependent graph was obtained for two types of *DNA origami* (4 and 12) at two different magnesium concentrations (Fig. 4c). Here we can observe the influence of the salt concentration in the solution but no influence of the number of probe strands per *DNA origami*, which suggests that the influence in the signal intensity is much more sensitive to the salt concentration and the hybridisation temperature rather than the number of sites where the reporter can bind with the target. Also further efforts to influence the binding strength by varying the probe numbers did not show conclusive results (Fig. S-2). Nevertheless, improvement of the hybridisation efficiency (or binding strength) by adjusting the salt concentration increases the signal-to-noise ratio.

**Figure 5:**
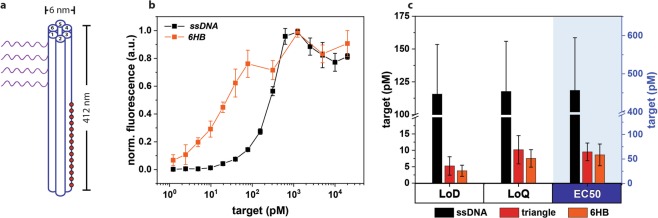
Six-helix bundle DNA origami as a reporter (*6HB*). (**a**) Schematic representation of a *6HB* with four probe strands (purple) and 14 ATTO 647N dye molecules (red circles). (**b**) Normalised surface fluorescence signal on microbead for *ssDNA* (black squares) and *6HB* (orange squares) for target sequence 2, with each reporter at 2.5 nM. (**c**) Averaged assay parameters (LoD, LoQ, EC50) determined from all five target sequences for all three types of reporters (black: *ssDNA* red: *DNA origami*, orange: *6HB*).

### Using *DNA origami* with a different shape

A six-helix bundle (*6HB*) DNA origami nanostructure^47^ was chosen as a different DNA origami reporter, and target response curves were obtained for the five different sequences as was used in Fig. 3. The *6HB* has a length of 412 nm and a diameter of 6 nm, with four probe strands and 14 dye molecules (Fig. 5a). The distance between each probe strand as well as the distance between the dye molecule handles were kept at 11–12 nm and 22 nm, respectively. As was shown by the triangular DNA origami reporter, using *6HB* showed a clear shift of the curves towards the lower target concentration regions (Fig. 5b). Averaged assay parameters of all five sequences for each type of reporter were obtained and are shown in Fig. 5c. The assay parameters for both shapes of DNA origami reporters were shown to be similar, a comparatively lower value than that of the ssDNA. For an example, the LoD was 5.2 pM and 3.8 pM for the triangular and 6HB *DNA origami*, respectively.

The clear distinction between *ssDNA* and DNA origami nanostructure based reporters are therefore confirmed. However, as was the case for *DNA origami*, the *6HB* showed much lower signal intensities (Fig. S-5). This is yet another clear indication of the complex binding behaviour at the surface of the microbead that deserves further investigation.

## Conclusions

In this work, we have put efforts into developing an improved reporter system to detect target oligonucleotides for microbead-based assays by using DNA origami nanostructures that are spatially addressable. Utilising this aspect of the *DNA origami*, 14 dye molecules were placed on each structure as well as a number of probe strands (ranging from 4 to 12) that could hybridise to the target oligonucleotide. VideoScan technology was used to measure the fluorescence signals of the assay, allowing for simple sample preparation as well as straightforward data collection. A target response curve was obtained comparing the two types of reporter, *ssDNA* and the new reporter *DNA origami*. This new reporter showed a better response to lower target oligonucleotide concentrations than the simple *ssDNA*, with lower values for all assay parameters while the dynamic range was wider. Multiplexed analysis was tested out by assigning each target sequence to a specific microbead population, which was encoded with a specific ratio of dyes. Attempts were also made to enhance the signal measured on the surface of the microbead by looking at the binding strength of the reporters to the targets, with higher ionic strength in the hybridisation solution showing higher fluorescence signals. The potential of increasing the binding strength by increasing the number of sites for the *DNA origami* to bind to the targets requires more in-depth studies in the future.

The findings show that this rationally designed reporter could lead to lower detection limits for oligonucleotides. The determined limit of detection was comparable to previously reported values in the femtomolar region^32,48,49^. It was however also apparent that the hybridisation of this reporter to the microbeads within this detection scheme can still be optimized. The measured surface fluorescence was not as high as anticipated, especially given the fact that one reporter structure carried 14 dye molecules. This indicated that the surface coverage of the microbead with the *DNA origami* was not optimal, and therefore the process of hybridisation of the reporter to the target was not as simple. This strategy still has many avenues for improvement, including optimizing the shape of the DNA origami nanostructures, e.g. more compact three dimensional structures^50^. The application of the 6HB reporter showed an indication of the target response curves being affected by the shape of the DNA origami nanostructure and therefore further studies to optimize the shape will be necessary. Utilising the potential of such programmable nanostructures as reporters could lead to highly sensitive and at the same time highly versatile assays. This reporter is also expected to translate well to detecting miRNA as targets, especially if the target capture strands are both RNA sequences which makes the oligonucleotide duplex more stable^37^.

## Methods

### DNA origami fabrication

DNA origami nanostructures were prepared according to previously described methods. The viral strand M13mp18 (tilibit nanosystems) was mixed with staple strands (unmodified) as well as staple strands extended at their 3′-end with T_40_ (probe strands) and dye labelling strands extended at their 5′-end with (AAT)_7_ in 10x concentrated TAE buffer containing 150 mM MgCl_2_ and ultrapure water (Merck Millipore). The solution was then annealed from 80 °C and slowly cooled down to 8 °C in 2 hours using a thermal cycler (PEQLAB/VWR). Using centrifugal filters (100kDA MWCO, Merck Millipore), the solution was washed 3 times (6000 rpm, 7 mins) with a buffer containing 1x TAE and 15 mM MgCl_2_ to remove excess staple strands. The subsequent hybridisation step to place dye molecules on the DNA origami nanostructure was performed by mixing the DNA origami solution with 5′-ATTO647N-(ATT)_7_ (Metabion) in buffer containing 1x TAE and 15 mM MgCl_2_. This solution was heated and kept at 45 °C for 41 mins and cooled down to 25 °C over 30 mins. This solution was then washed again with the centrifugal filters to remove excess dye modified oligonucleotides (6 times, 6000 rpm, 7 mins). The concentration of the DNA origami solution was confirmed by using UV-vis absorption spectroscopy (NanoDrop 2000, Thermo Scientific). For more details including the sites with modification for probe strands and dye placement, see Supplementary Information (Table S-1).

### AFM imaging

Confirmation of the folded triangular DNA origami nanostructures was done by AFM imaging. 2 μL of the purified sample was adsorbed on freshly cleaved mica (Plano) wih 28 μL of buffer (1x TAE with 15 mM MgCl_2_). After incubating for 2 mins, the sample was washed with 3 mL ultrapure water and dried immediately with compressed air. Imaging was conducted under dry conditions (Flex AFM, Nanosurf) using cantilevers with a frequency of 150 kHz and a spring constant of 5 Nm^−1^ (Tap 150 Al-G, Budget Sensors).

### Sample preparation

Microbeads (size range 11.5–14 μm) stained with two fluorescent dyes (PolyAn GmbH), or encoding dyes, with different ratios were prepared according to previously described methods^36^. Streptavidin coated microbeads were loaded with biotinylated oligonucleotides complementary to the target sequence (capture oligonucleotide). Subsequently, the target oligonucleotides that were extended by A_40_ were incubated with the functionalised microbeads for 1 hour at room temperature in a shaker. After carefully removing excess solution from the microbead solution after centrifugation (2250 *g*, 3 min), the pellets were resuspended in 10 μl of either *ssDNA* or *DNA origami* solution of which the concentration was adjusted to 2.5 nM. After incubating for 1 hour at room temperature, the samples were kept at 4 °C overnight. For more details including DNA sequences of capture and target strands see Table S-2. A more detailed description of microbead preparation is given in the supplementary information.

### Data acquisition using VideoScan technology

The sample solution was transferred to 96-well plates, and the fluorescence signal was detected using the VideoScan technology^32^ after the microbeads had settled to the bottom (10 mins). Briefly, the microbeads were first identified using two filter sets for the encoding dyes and the ratio between the two dyes was determined. Subsequently, a third set of filters was used to measure the analyte label (ATTO 647n) on the surface of the microbead.

### Determination of assay parameters

Assay parameters were determined according to the following equations,

$${\rm{LoD}}=(3.29\times {\sigma }_{0}+B)/A,$$

$${\rm{LoQ}}=(10\times {\sigma }_{0}+B)/A,$$

$${\rm{EC50}}=[({y}_{max}+{y}_{{\rm{\min }}})/2+B]/A,$$

where σ_0_ is the standard deviation of the blank, *A* is the slope and *B* the y intercept of the calibration curve. *y*_*max*_ is the maximum and *y*_*min*_ the minimum measured fluorescence intensity of the assay. Calibration curves are determined for the region between LoQ and limit of linearity, which is the region where the response of the fluorescence to the target oligonucleotide concentration is linear.

## Supplementary information

Supplementary Information

## Data Availability

The datasets generated during and/or analysed during the current study are available from the corresponding author on reasonable request.
