# Supplementary material for: A new reporter design based on DNA origami nanostructures for quantification of short oligonucleotides using microbeads
Source: Sci Rep. 2019 Mar 18;9:4769. doi: 10.1038/s41598-019-41136-x (PMC6423227; doi:10.1038/s41598-019-41136-x)
Supplement: Supplementary file 1 — Supplementary Information [file 41598_2019_41136_MOESM1_ESM.pdf]

Supplementary information

A new reporter design based on DNA origami nanostructures for quantification of short oligonucleotides using microbeads.

Youngeun Choi[1,2], Carsten Schmidt[3], Philip Tinnefeld[4], Ilko Bald[1,2*], Stefan Rödiger[3*]

[1]University of Potsdam, Department of Chemistry, Physical Chemistry, 14476 Potsdam, Germany

[2]BAM Federal Institute for Materials Research and Testing, 12489 Berlin, Germany

[3]Brandenbrug University of Technology Cottbus-Senftenberg, Institute of biotechnology, 01968 Senftenberg, Germany

[4]Department Chemie and Center for NanoScience, Ludwig-Maximilians-Universitaet Muenchen, Butenandtstr. 5-13 Haus E, 81377, Muenchen, Germany

*Email : stefan.roediger@b-tu.de, bald@uni-potsdam.de

1. Staple strand modification for *DNA origami* design

Each staple oligonucleotide is addressable, giving the DNA origami staple control with high versatility.

DNA origami	*Probe strands*<br>3′- end elongated with T_{40}	*dye label handles*<br>5′- end elongated with $(AAT)_7$
**4** 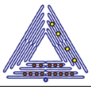	t-6s17f, t-2s17f, t 2s17f, t 6s17f	t-7s8g, t-5s8g, t-3s8g<br>t-3s4e, t-1s8g, t-1s4e<br>t 1s4i, t 1s8i, t 3s4e<br>t 3s8g, t 5s4e, t 5s8g<br>t 7s8g, t 9s8g<br>Further hybridisation with 5′-ATTO 647N-(ATT)7
**4-2** 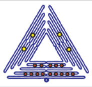	t-2s17f, t 2s17f<br>t-2s27f, t 2s27f	
**6** 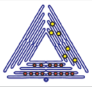	t-6s17f, t-4s15f, t-2s17f<br>t 6s17f, t 4s15f, t 2s17f	
**6-2** 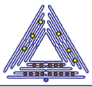	t-6s17f, t-4s15f, t 2s17f<br>t-6s27f, t-2s27f, t 2s27f	
**8** 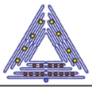	t-6s17f, t-2s17f, t 2s17f, t 6s17f<br>t-6s27f, t-2s27f, t 2s27f, t 6s27f	
**10** 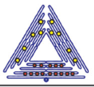	t-6s17f, t-2s17f, t 2s17f, t 4s15f, t 6s17f<br>t-6s27f, t-4s25f, t-2s27f, t 2s27f, t 6s27f	
**12** 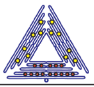	t-6s17f, t-4s15f, t-2s17f, t 2s17f, t 4s15f, t 6s17f<br>t-6s27f, t-4s25f, t-2s27f, t 2s27f, t 4s25f, t 6s27f	

Table S-1. Probe strands and dye label handle positions in the design of the DNA origami, according to the number/position of the probe strands. Staple names and sequences are taken from the Rothemund triangle design[1]. Elongated staple strands act as sticky ends for further hybridisation steps such as binding dye labeled DNA for labeling the *DNA origami* or binding to the A_{40} part of the target oligonucleotide.

By elongating or labeling the staple strands, either at the 3′- or the 5′- end, at positions where the modifications are required the required functionality can be incorporated into the design. Positions of elongated staple strands for target binding (*probe strands*) and handles for hybridising dye-labeled DNAs (*dye label handles*) are shown in Table S-1. The sequence and nomenclature have been used as was reported previously by Rothemund[1]. It is also worth noting that by having dye label handles

instead of having staples at the positions each modified by ATTO 647N, costs can be cut down as only one sequence that is labeled with the dye is required for the 14 modifications.

2. Microbead-based assay

The fluorescence signal measured from the microbead assay is suboptimal when using *DNA origami* nanostructures as reporters. The signal intensity is approximately 10 fold weaker than when using *ssDNA* as the reporter with only one dye per reporter (Fig S-1.).

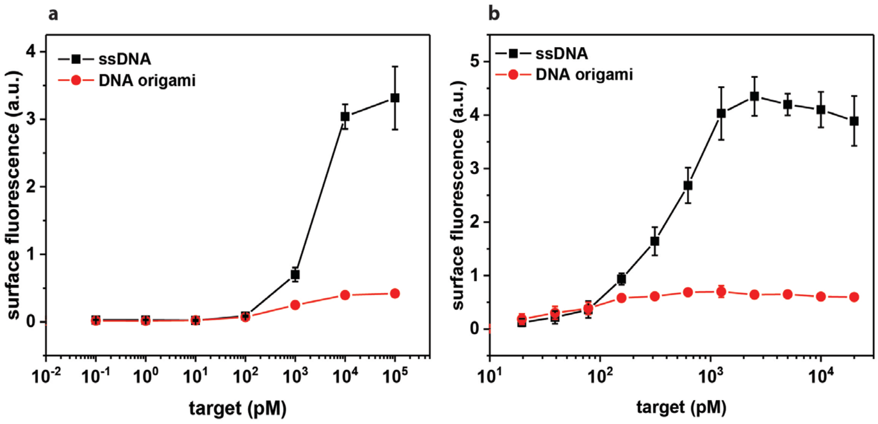

Figure S-1. Fluorescence signal measured by videoscan before intensity normalization for *ssDNA* (■) and *DNA origami* (●). (A) Fluorescence signals obtained or the target concentration between 0.1 pM to 100 nM and (B) for a zoomed-in range of target concentration of the dynamic range.

3. Proof-of-concept multiplex analysis

Target oligonucleotides were designed to be a proof-of-concept multiplex analysis using a novel reporter designed based on DNA origami nanostructures. As targets, we chose DNA equivalents of human miRNAs that have already been discovered and are used as biomarkers in profiling [2]. DNA sequences used as target oligonucleotide, biotin modified target capture oligonucleotide are listed in Table S-2.

**Target oligonucleotide**			
name	Corresponding miRNA	Target DNA sequence	Human miRNA seqeunce
Target 1	has-miR-21	TAGCTTATCAGACTGATGTTGAA$_{40}$	uagcuuaucagacugauguuga
Target 2	hsa-miR-106a-5p	AAAAGTGCTTACAGTGCAGGTAGA$_{40}$	aaaagugcuuacagugcagguag
Target 3	hsa-miR-4484	AAAAGGCGGGAGAAGCCCCAA$_{40}$	aaaaggcgggagaagcccca
Target 4	hsa-miR-15a-5p	TAGCAGCACATAATGGTTTGTGA$_{40}$	uagcagcacauaaugguuugug
Target 5	hsa-miR-486-5p	TCCTGTACTGAGCTGCCCCGAGA$_{40}$	uccuguacugagcugccccgag

**Target capture oligonucleotide**		
name	Capture DNA sequence	Spacer (3'-end of DNA)
Capture-Target1	TCAACATCAGTCTGATAAGCTA	-HEGL-Biotin
Capture-Target2	CTACCTGCACTGTAAGCACTTTT	

Capture-Target3	TGGGGCTTCTCCCGCCTTTT	
Capture-Target4	CACAAACCATTATGTGCTGCTA	
Capture-Target5	CTCGGGGCAGCTCAGTACAGGA	

Table S-2. DNA oligonucleotide sequences used for proof-of-concept miRNA detection scheme.

4. Effect of increasing the number of T40- elongated staple strands

A number of *DNA origami* samples were prepared with varying number of probe strands ranging from 4 to 12 with the closest neighbouring probe strands being 12.7 nm apart. In addition, for the reporters with four and six probe strands, the strands were either arranged on one side of the triangle or on two sides of the triangle (Fig. S-2 a). When the *DNA origami* had only one probe strand, no fluorescence signal was detected emphasising the importance of the binding strength in detecting the targets (data not shown). Fig. S-2 b shows the fluorescence measured for each type of the *DNA origami*, with the concentration of the reporter and the target oligonucleotide consistent for all samples. The correlation between the numbers of probe strands to the measured fluorescence is not apparent from this graph and therefore a final conclusion regarding this particular aspect, the effect of binding strength of the reporter to the microbead immobilised target oligonucleotide, cannot be drawn.

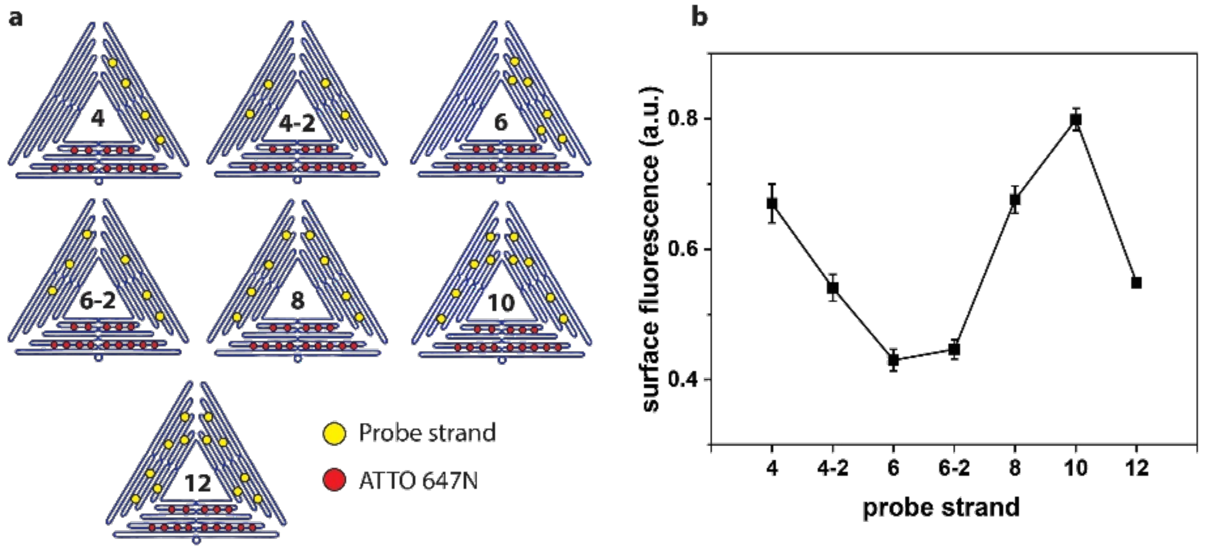

Figure S-2. Binding strength effects. **a** Schematic representation of the DNA origami with different number of probe strands ranging from 4 to 12 with 4-2 and 6-2 with probe strands distributed on two sides of the triangle instead of just one side. **b** Surface fluorescence measured after hybridisation of the reporters on the microbeads according to the number of probe strands (target oligonucleotide was fixed to 20 nM and *DNA origami* to 2.5 nM).

5. Comparison of different DNA origami nanostructures as reporter

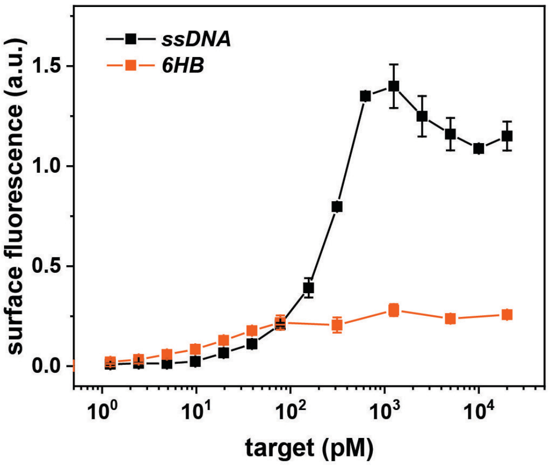

Figure S-5. Fluorescence intensity measured by videoscan using *ssDNA* (■) and *6HB* (■). The surface intensities measured using *6HB* is significantly lower than that of *ssDNA*, confirming the complex binding behaviour of the reporter at the surface of the microbeads.

6. Sample preparation (microbead)

Streptavidin was generated recombinantly according to Gallizia et al[3]. Briefly, we produced streptavidin with *E. coli* (BL21DE3 pLys) transformed with a pET11a containing the streptavidin DNA. Cells were disrupted with french pressing, cell debris were removed by centrifugation and the supernatant was incubated with 2-iminobiotin-sepharose (Sigma). Sepharose was washed away repeatedly until the absorption at 260 nm reached the baseline level. Finally streptavidin was eluted with 1% acetic acid. By using centrifugal filter units (Millipore), acetic acid was exchanged by diluted PBS and the solution was concentrated (1 mg/mL streptavidin).

Preparation of streptavidin coupled microbeads (11.5 – 14 µm) were done as previously described by Rödiger et al[4]. Briefly, 150000 fluorescence encoded carboxylated polymethylmethacrylate microbeads (PolyAn GmbH, Berlin, Germany) were activated with 25 mg mL^{-1} N-(3-dimethylaminopropyl)-N'-ethylcarbodiimide hydrochloride (EDC, Sigma, St-Louis, MO, USA) dissolved in 0.1 M 2-(N-morpholino)ethanesulfonic acid buffer (pH 4.5) (MES, Sigma, St. Louis, MO, USA) for 30 min at 28°C. After activation, excessive EDC was removed by washing the microbeads with 200 µl diluted PBS (2.5 mM Na-phosphate pH 7.4, 7.5 mM NaCl). The activated microbeads were then incubated in 50 µl of 150 µg/mL streptavidin dissolved in diluted PBS-buffer for 3 h at 28°C. Excessive streptavidin was removed with three washing steps using 200 µl TBST (50 mM Tris/HCl (pH 7.5), 154 mM NaCl, 0.01% Tween-20).

200 µl of 100 nM biotinylated capture probe was given to 50000 streptavidin coupled microbeads. After incubation for 1 h at 37°C the microbeads were washed three times with 200 µl hybridisation buffer (Hy-buffer: 67 mM Tris/HCl (pH 8,5), 16 mM $(NH_4)_2SO_4$, 15 mM $MgCl_2$,0.01% Tween-20).

For hybridisation analysis of target oligonucleotides, the desired concentrations of the targets were prepared using Hy-buffer. 100 µl of this concentration adjusted target oligonucleotide solution was given to a microbead mixture containing approximately 100 microbeads per population. After one hour of incubation at 25°C, the samples were kept at 4 °C overnight before unbound oligonucleotides were removed by washing the microbeads three times with 200 µL Hy-buffer (2250 *g*, 3 min). It must be noted here that the mixture of microbead population had to be hybridised with only one target oligonucleotide. In fact, if the microbead population mixtures were exposed to more than one sequence of the target oligonucleotide there was no fluorescence signal that could be measured (data not shown). This indicated that the hybridisation of these oligonucleotides to the capture strands

functionalised on the surface of the microbead is not straightforward, and many competing factors exist.

For multiplex analysis, different microbead populations presenting different capture probes on their surfaced were mixed together before measurements.

References

1. Rothemund, P. W. K. Folding DNA to create nanoscale shapes and patterns. *Nature* **440,** 297–302; 10.1038/nature04586 (2006).
2. Sauer, E., Reinke, A.-K. & Courts, C. Differentiation of five body fluids from forensic samples by expression analysis of four microRNAs using quantitative PCR. *Forensic science international. Genetics* **22,** 89–99; 10.1016/j.fsigen.2016.01.018 (2016).
3. Gallizia, A. *et al.* Production of a soluble and functional recombinant streptavidin in Escherichia coli. *Protein expression and purification* **14,** 192–196; 10.1006/prep.1998.0930 (1998).
4. Rödiger, S. *et al.* Fluorescence dye adsorption assay to quantify carboxyl groups on the surface of poly(methyl methacrylate) microbeads. *Analytical chemistry* **83,** 3379–3385; 10.1021/ac103277s (2011).
